# Direct blood fluorescence signal intensity of neutrophils (NEU-SFL): A predictive marker of death in hospitalized COVID-19 patients?

**DOI:** 10.3389/fmed.2022.1062112

**Published:** 2022-12-21

**Authors:** Mathieu Fortier, Mathias Chea, Charlène Aïn, Maxime Loyens, Thierry Boudemaghe, Jean-Christophe Gris, Sylvie Bouvier

**Affiliations:** ^1^Department of Hematology, University Hospital, Nîmes, France; ^2^UA11 INSERM – UM Institut Desbrest d’Épidémiologie et de Santé Publique (IDESP), Montpellier, France; ^3^Department of Medical Information, Methods and Research, Centre Hospitalier Universitaire de Nîmes, University of Montpellier, Nîmes, France; ^4^I.M. Sechenov First Moscow State Medical University, Moscow, Russia; ^5^Faculty of Pharmaceutical and Biological Sciences, Montpellier University, Montpellier, France

**Keywords:** COVID-19, fluorescence signal intensity, immunothrombosis, NETosis, neutrophil

## Abstract

**Introduction:**

Coronavirus disease 2019 (COVID-19) is a respiratory disease triggered by immunopathological mechanisms that cause excessive inflammation and leukocyte dysfunction. Neutrophils play a critical role in the innate immunity and are able to produce neutrophil extracellular traps (NETs: NETosis process) to combat infections. Some NETs markers are increased in patients who died from COVID-19. Recently, the neutrophil fluorescence variable (NEU-SFL), available on certain automated complete blood count (CBC) analyzers, has been correlated with NET formation and may reflect NETosis in patients. Here we evaluate whether NEU-SFL measured after admission of COVID-19 patients is associated with in-hospital survival or death.

**Patients and methods:**

1,852 patients admitted for severe COVID-19 at Nîmes University Hospital in 2021 were retrospectively included in the study: 1,564 who survived the hospital stay and 288 who did not. The NEU-SFL was obtained on the Sysmex™ XN-10^®^ analyzer and values for survivors and non-survivors were compared. The intra-patient NEU-SFL variations between the hospital entry and the last day of hospitalization were also analyzed (IRB 22.06.01, NCT 05413824).

**Results:**

Non-survivors presented higher NEU-SFL values. NEU-SFL values above the 4th quartile were independently associated with a 2.88-fold risk of death. Furthermore, the difference of NEU-SFL values between the first and the last available data during hospitalization revealed that a decrease in NEU-SFL was associated to survivors and vice versa.

**Conclusion:**

Our study reinforces the role of neutrophils and NETosis in the pathophysiology and prognosis of COVID-19. Further studies combining NEU-SFL with other NETosis markers could improve the management of COVID-19 patients.

## Introduction

Severe acute respiratory syndrome coronavirus 2 (SARS-CoV-2) infection, initially described in 2019 in China, quickly spread to become a global pandemic. An association between coronavirus disease 2019 (COVID-19) and coagulation test abnormalities, such as an increase in D-Dimers (1), as well as the occurrence of thrombotic events (2), is widely described. This increased thrombotic risk is understood to be linked to the mechanism of “thromboinflammation,” a process by which the innate immune system and the inflammation caused by the viral infection activate coagulation at the origin of severe coagulopathies (3). On the other hand, in post-mortem examinations on COVID-19 patients, extravasation of neutrophils—key cells in innate immunity—has been widely observed in the pulmonary capillaries, myocardium and liver (4).

Formation of neutrophil extracellular traps (NETs), process called NETosis, is a particular form of cell death in neutrophils. It is characterized by the release of DNA, histones and antimicrobial enzymes in the form of filaments called “NETs” (5). NETosis is induced by various factors including microbial and pro-inflammatory stimuli. A dysregulated generation of NETs and the process of NETosis have been described in many COVID-19 patients (6). In addition, a recent study has shown that indirect markers of NETosis, such as cell-free DNA (cf DNA), myeloperoxydase (MPO)-DNA complexes, and citrullinated histone H3, are higher in non-surviving COVID-19 patients than in surviving patients (7).

These methods of analysis being time-consuming, some studies have recently focused on the neutrophil side fluorescence light index (NEU-SFL), which is systematically quantified by the XN-10^®^ complete blood count (CBC) automated analyzer from Sysmex (Sysmex™ Corporation, Kobe, Japan). This index is generated by incorporating fluorescent dye which targets the de-condensed DNA of permeabilized neutrophils (8). Interestingly, it was found that the NEU-SFL index increased in patients with septic shock complicated by intravascular coagulation (DIC). The study of Stiel and collaborators also showed that the increase of NEU-SFL was positively correlated with NETs formation, suggesting that the NEU-SFL index could be considered as an indirect marker of NETosis (9–11). Another recent study describes a higher NEU-SFL index in patients with severe COVID-19 syndrome, but this parameter did not discriminate patients with or without distal vein thrombotic complications (12).

In order to characterize a predictive marker of death by COVID-19 faster to collect that can improve medical decision, we simply evaluated the NEU-SFL index of patients admitted to Nîmes University Hospital for severe COVID-19 in 2021, depending on their survival status during their hospital stay.

## Methods

### Study design and participants

We made a retrospective cohort study using the national “Programme de médicalisation des systèmes d’information” (program for the medicalization of information systems = PMSI) database, designed to include discharge summaries of all patients admitted to hospitals in France.

For the COVID-19 cohort in this study, all adult patients admitted to Nîmes University Hospital for COVID-19 from January 1 to December 31, 2021, were included. Hospital admissions for COVID-19 were identified by primary diagnoses, related diagnoses, or associated diagnoses, with ICD-10 codes U07.10, U07.11, U07.14, or U07.15.

The study was approved by the Institutional Review Board (IRB 22.06.01) and ethics committee at Nîmes University Hospital. This clinical investigation was performed in accordance with the Helsinki declaration of 1975 as revised in 1996. A non-opposition letter was sent to all patients and only those who refused to participate were excluded (NCT 05413824).

The outcomes of all patients admitted for COVID-19 (survival or death) and their respective hospitalized duration, if any, were collected.

### Blood cell count and neutrophil analysis

For each participant, 5 mL of blood was drawn into EDTA [ethylene-diamine-tetra-acetic acid dipotassium salt dihydrate (EDTA-2K) anticoagulant] collection tubes (BD Vacutainer Tubes, Becton Dickinson, Le Pont de Claix, France). A complete blood cell count was made at admission on an automated Sysmex™ XN-10^®^ analyzer (Sysmex Corporation, Kobe, Japan) according to the manufacturer’s instructions. Further blood cell counts were performed if the patient remained hospitalized for several days. Side Fluorescence Light from neutrophils (NEU-SFL), described as reflecting neutrophil activation (9), was extracted from the research screen of the analyzer’s software. The NEU-SFL index was obtained after cell permeabilization by a specific Sysmex lysis reagent, allowing the XN-10 basic fluorescent polymethine dye to enter the cells. This dye binds to nucleic acids in the cytoplasmic organelles and the nucleus (9). Other biological markers (D-Dimers, fibrinogen, C reactive protein) were assayed as previously described (13, 14).

### Statistical analysis

Data were analyzed with version 5.01 of the GraphPad^®^ statistical software program (San Diego, United States) using Mann-Whitney, Wilcoxon, Fisher’s and chi-square tests for two group comparisons as appropriate. ROC curve and logistics regression were made using Stat View^^®^^ (Abacus concepts, Berkeley, CA, USA).

The associations between NEU-SFL values and the patient outcome were explored by means of logistic regression analysis and estimated from odds ratios (OR) with a 95% confidence interval (CI). For these analyses, patients were divided into quartiles for NEU-SFL values with the lowest quartile (≤25th percentile) used as the reference. The potential confounding influence of simple demographic, laboratory risk factors for death due to COVID-19 (i.e., age, gender, blood cell count, and inflammation parameters) and comorbidities on the associations between NEU-SFL and patient outcomes were evaluated using multivariate logistic regression models. Only biological parameters with less than 10% missing data were integrated into the model.

We also studied the absolute difference between the first (entry in hospital) and last available NEU-SFL values (Delta NEU-SFL) in patients whose hospital stay had lasted at least 5 days. The strength of concordance between Delta NEU-SFL and survival was evaluated by C statistics, calculating the area under the receiving operating curve (AUROC). The best discriminating point was evaluated with the Youden index and its corresponding positive predictive value (PPV) and negative predictive value (NPV) were computed.

Quantitative data were expressed as medians with interquartile ranges [Q1; Q3]. Qualitative data were expressed as absolute numbers and frequencies (%). A *p*-value < 0.05 was considered as statistically significant.

## Results

### Participants

**FIGURE 1:**
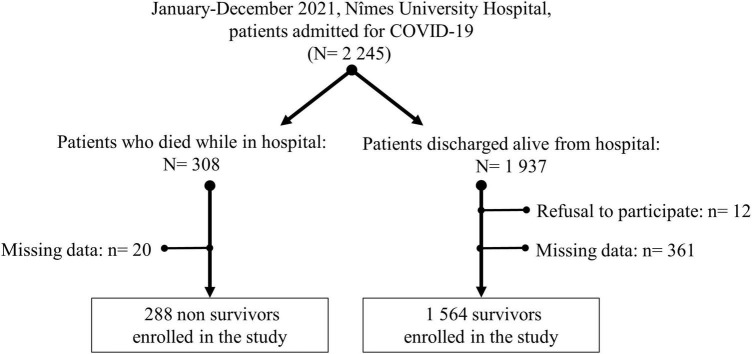
Participant flowchart.

In 2021, 2,245 adult patients were admitted to Nîmes University Hospital for severe COVID-19 and 1,852 patients were finally enrolled in the study: 1,564 who finally survived their hospital stay (survivor group) and 288 who unfortunately did not (non-survivor group). Reasons for non-inclusion were mainly missing data and very few patients were unwilling to participate. The flowchart is detailed in Figure 1 and characteristics of the population are summarized in Table 1. As the medians of ages were significantly different (*p* < 0.0001) between survivors and non-survivors, we checked to see whether the NEU-SFL increase in patients who died was not biased due to age. We observed no correlation between NEU-SFL and age (*p* = 0.15) (data not shown).

**TABLE 1:** Population characteristics.

	Non-survivors	Survivors	*P*
**N**	288	1 564	
Gender			0.22
Male, n (%)	169 (59%)	857 (55%)	
Female, n (%)	119 (41%)	707 (45%)	
Age, years	82 [74–88]	65 [52–75]	** < 0.0001**
Intensive care unit	50 (17%)	215 (14%)	0.11
**Comorbidities n (%)**			
No comorbidity	50 (17%)	766 (49%)	** < 0.0001**
Hypertension	131 (45%)	490 (31%)	** < 0.0001**
Diabetes mellitus	96 (33%)	335 (21%)	** < 0.0001**
Chronic lung disease	37 (13%)	136 (9%)	**0.03**
Chronic heart disease	47 (16%)	90 (6%)	** < 0.0001**
Chronic kidney disease	59 (20%)	120 (8%)	** < 0.0001**
Cancer	44 (15%)	82 (5%)	** < 0.0001**
Leukocytes, 10^9^/L	7.34 [5.29; 10.43]	6.66 [4.93; 9.07]	**0.004**
Neutrophils, 10^9^/L	5.84 [3.93; 8.63]	4.88 [3.41; 7.22]	** < 0.0001**
Lymphocytes, 10^9^/L	0.81 [0.53; 1.11]	0.99 [0.68; 1.41]	** < 0.0001**
Monocytes, 10^9^/L	0.42 [0.25; 0.73]	0.47 [0.31; 0.68]	0.06
Hemoglobin, g/L	127 [107.3; 140]	134 [121; 146]	** < 0.0001**
Platelets, 10^9^/L	183 [149; 264]	210 [163; 282]	**0.0002**
Prothrombin time, %, (n)	85 [65; 100] (217)	97 [87; 100] (1,238)	** < 0.0001**
Fibrinogen, g/L, (n)	6.20 [5.09; 7.02] (130)	6.26 [5.27; 7.40] (675)	0.22
D-dimers, ng/mL, (n)	1,585 [895; 2,835] (144)	995 [655; 1,623] (978)	** < 0.0001**
C reactive protein, mg/L, (n)	80.50 [43.65; 150.60] (285)	57.35 [20.73; 119.30] (1508)	** < 0.0001**

Results are represented as medians with their interquartile range [Q1; Q3]. For parameters with missing data, the number of patients analyzed is indicated in brackets. Bold values represented the significant data.

### NEU-SFL values and patient outcomes

**FIGURE 2:**
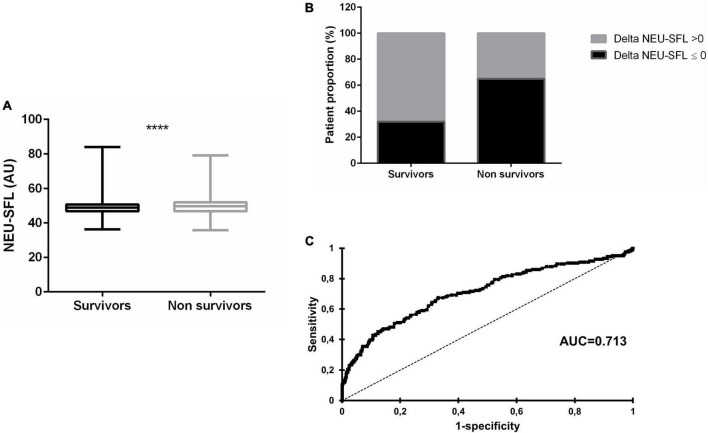
Fatal COVID-19 is associated with initially higher and increasing NEU-SFL values during hospitalization. **(A)** Boxplot representation of NEU-SFL at admission in survivors (*n* = 1 564) and non-survivors (*n* = 288). Results are represented with medians and ranges. AU, arbitrary unit. **(B)** Comparison of Delta NEU-SFL distribution between the hospital entry and the last day of hospitalization in hospitalized COVID-19 survivors and non-survivors. For positive Delta NEU-SFL values, *n* = 385 for survivors and *n* = 58 for non-survivors. For null or negative Delta NEU-SFL values, *n* = 181 for survivors and *n* = 108 for non-survivors. A positive delta NEU-SFL corresponds to a decrease in NEU-SFL values between admission and the last day of hospitalization; a negative delta-NEU-SFL corresponds to an increase in NEU-SFL between admission and the last day of hospitalization. **(C)** Receiver operating characteristic curve between Delta NEU-SFL and in-hospital survival. Delta NEU-SFL: absolute difference between the NEU-SFL index value at admission and the last available one. AUC, area under the curve. ****p < 0.0001.

NEU-SFL values at admission were significantly (but only slightly) higher in non-survivors than in survivors (Figure 2A). Median levels of NEU-SFL in non-survivor patients were 49.60 AU [46.73; 52.00] vs. 48.8 AU [46.70; 50.70] in survivors (*p* = 0.005).

After categorization of NEU-SFL values, patients belonging to the 4th quartile of the distribution (i.e., with a NEU-SFL quantification result higher than 51 AU) had a higher risk of death than patients with values in the first percentile (OR, 1.49; 95% CI, 1.07–2.10, *p* = 0.02). After adjusting for potential confounders with less than 10% of missing data (i.e., age, gender, leukocytes and neutrophils count, hemoglobin, platelets, C reactive protein) and also comorbidities (hypertension, diabetes mellitus, chronic heart diseases, chronic lung diseases, chronic kidney disease, cancer), NEU-SFL values belonging to the 4th quartile of its distribution remained an independent predictor of death (adjusted OR, 2.88; 95% CI, 1.86–4.46, *p* < 0.0001) (Table 2), together with increasing ages and male sex.

**TABLE 2:** Association between NEU-SFL index values at admission and death of patients with COVID-19 considering the potential confounding influence of demographic, laboratory risk factors available for at least 90% of the patients and comordidities.

	Adjusted odds ratio (95% CI)	*P*
NEU-SFL quartile Q1:	1.00	
NEU-SFL quartile Q2:	1.03 (0.65; 1.64)	0.89
NEU-SFL quartile Q3:	1.02 (0.63; 1.63)	0.95
NEU-SFL quartile Q4:	2.88 (1.86; 4.46)	**<10^–4^**
Age, years	1.1 (1.08; 1.12)	**<10^–4^**
Gender: male	1.48 (1.07; 2.06)	**0.0168**
Hypertension	1.00 (0.7; 1.44)	0.99
Diabetes mellitus	0.72 (0.47; 1.10)	0.125
Chronic lung disease	1 (0.6; 1.67)	1
Chronic heart disease	0.98 (0.55; 1.75)	0.94
Chronic kidney disease	1.23 (0.72; 2.09)	0.44
Cancer	0.93 (0.43; 1.99)	0.85
Other diseases	0.73 (0.46–1.17)	0.191
Hemoglobin, g/L	1.01 (0.92; 1.09)	0.87
Platelets, 10^9^/L	1.001 (0.999; 1.002)	0.51
Leukocytes, 10^9^/L	0.98 (0.94; 1.02)	0.40
Neutrophils, 10^9^/L	1.002 (0.987; 1.017)	0.82
C Reactive Protein, mg/L	0.998 (0.996; 1.001)	0.19

Adjustment was computed on all the variables in the table. CI, confidence interval. Bold values represented the significant data.

### NEU-SFL values and patient outcomes according to their transfer or not in intensive care units

The comparison of NEU-SFL between patients who necessitated transfer to intensive care units (ICUs) with those who did not, showed that patients who necessitated transfer to ICUs had higher values of NEU-SFL (*p* = 0.0009). Furthermore, when we compare NEU-SFL values in survivors and non-survivors according to their passage or not in ICUs, we found a significant difference only in patients who were not transferred to ICUs (*p* = 0.0017) (data not shown).

### Comparison of NEU-SFL between the hospital entry and the last day of hospitalization

We then analyzed NEU-SFL variations between the first and the last available day of hospitalization, for a stay of at least 5 days, in the subgroup of patients for whom these data were available (*N* = 732; survivors: *N* = 566; non-survivors: *N* = 166). We calculated the absolute difference between the first and last available NEU-SFL values (Delta NEU-SFL). Interestingly, the majority of the survivors had a positive difference in NEU-SFL over the course of hospitalization (i.e., a lower value at the end of the stay), but the majority of non-survivors had a negative difference, thus a higher NEU-SFL value at the end of their hospital stay (Figure 2B). Fisher’s exact test highlighted a strong significant difference between the two groups (*p* < 0.0001). The AUC computed by C statistics was 0.713 (95% CI, 0.657–0.769, *p* < 0.0001) (Figure 2C), indicating a good—but not excellent—concordance between Delta NEU-SFL and in-hospital survival. The best discriminating value was −0.2 with a corresponding PPV of 0.375 (95% CI, 0.338–0.412) and an NPV of 0.875 (95% CI, 0.848–0.898).

### Routine laboratory parameters and patient outcomes

Results of routinely drawn coagulation or inflammation-based laboratory and blood cell count parameters (prothrombin time, fibrinogen, D-dimers, C reactive protein, leukocytes, neutrophils, lymphocytes, monocytes, hemoglobin, and platelet count), were evaluated and compared between survivors and non-survivors in Table 1 of the manuscript. Leukocytes, neutrophils, d-dimers, and C reactive protein were higher in non-surviving patients than in survivors. On the other hand, lymphocytes, hemoglobin, and prothrombin time were lower in non-survivors than in survivors. Fibrinogen is commonly elevated in COVID-19 patients.

## Discussion

The major new finding of this study is that the direct blood fluorescence signal intensity of neutrophils (NEU-SFL) was higher in severe COVID-19 patients who died during their hospital stay than in patients who survived. NEU-SFL values above the 4th quartile were independently associated with a 2.88-fold risk of death after adjusting for potential confounders (demographic, laboratory risk factors, and comorbidities). Furthermore, the comparison of NEU-SFL values between those at hospital entry and the last laboratory assessment showed an association with survival/non-survival.

NEU-SFL signals are generated by incorporating a fluorescent dye that targets unpacked DNA within the permeabilized cell. NEU-SFL was recorded as a surrogate for DNA unpacking in the neutrophil and is proposed as an indicator for NETosis (9). Most studies on NEU-SFL and NETosis have shown a positive association between NEU-SFL and disseminated intravascular coagulation (DIC) (9). Coagulopathy is described in COVID-19 and constitutes an aggravating factor for infection (15). In COVID-19 patients, abnormal coagulation is common, and in most cases, DIC was observed in individuals with COVID-19 during hospitalization before they eventually died (16). This is in agreement with our results showing that NEU-SFL is higher in non-survivors and that NEU-SFL increases during hospitalization in patients with a poor outcome. This result concurs with the study by Dennison and collaborators, showing that the intensity of neutrophils’ reactivity (NEU-RI), which also measures neutrophil activation, is associated with poor outcome. However, their study was conducted on a small number of patients. Our work was carried out on a larger cohort, and our quartile categorization shows that only the 4th quartile reveals a positive odds-ratio (17). In addition, our study brings new elements on NEU-SFL variation during hospitalization stay, this value seeming more reliable as a predictive tool. The comparison of NEU-SFL values in survivors and non-survivors according to their passage or not in ICUs showed a significant difference only in patients who were not transferred to ICUs. This result is similar to the difference observed between all survivors and all non-survivors, confirming the fact that NEU-SFL increases in a severe and inflammatory-condition infection.

Furthermore, other indirect markers of NETosis have already been studied in patients with COVID-19 such as total DNA, myeloperoxidase (MPO)–DNA complexes, and citrullinated histone H3. These revealed an increase in circulating biomarkers for NETs in patients who died from COVID-19 and also in patients who subsequently developed thromboembolic complications (7). Other studies focused on *ex vivo* neutrophil activation, but these approaches are too time-consuming and inappropriate for routine analysis compared to a parameter obtained automatically from a CBC analyzer (18, 19).

Concerning the variations in routinely drawn coagulation or inflammation-based laboratory and blood cell count parameters, the variations between surviving and non-surviving patients are in agreement with what has been described in the literature (20). As expected, non-survivors presented higher rates of comorbidities. However, NEU-SFL remains statistically higher in non-survivors without comorbidity compared to survivors (*p* = 0.013), suggesting that comorbidities do not influence the NEU-SFL (data not shown) (21). This is confirmed by the results obtained in our multivariate analysis.

Our study has certain limitations. It is a single-center retrospective study with a considerable age difference between survivors and deceased patients. However, age is a well-known risk factor for the requirement of advanced medical care in COVID-19 patients (22), and we found that NEU-SFL and age were not correlated, the increase in NEU-SFL in non-survivors being unrelated to the age of patients. In addition, we did not have any information on the patients’ vaccination status. It might be interesting to compare NEU-SFL values in vaccinated vs. non-vaccinated patients.

Our study also has a strength. NEU-SFL has the great advantage of being easily, automatically and systematically available from a standard blood sample submitted for CBC and differential using commercially available automates, whereas other NETosis biomarkers are less easy to obtain. It is the first to investigate NEU-SFL in a large retrospective cohort of COVID-19 patients and to evaluate its variations during hospitalization.

To conclude, our results showed that fatal COVID-19 is associated with initially higher, and increasing NEU-SFL index values, once again supporting the role of neutrophils in the pathophysiology of COVID-19 with a poor outcome. However, we observed only slightly higher values in non-survivors, with a strong overlap of values between fortunate and unfortunate patients: NEU-SFL alone does not strongly discriminate the vital status. Further studies combining NEU-SFL with other NETosis or neutrophil activation markers, or with complementary biomarkers, could help clinicians improve management of the COVID-19 coagulopathy.

## Data availability statement

The original contributions presented in this study are included in the article/supplementary material, further inquiries can be directed to the corresponding author.

## Ethics statement

The studies involving human participants were reviewed and approved by the Institutional Review Board (IRB 22.06.01) and Ethics Committee at Nîmes University Hospital. Written informed consent for participation was not required for this study in accordance with the national legislation and the institutional requirements.

## Author contributions

MF designed the research, analyzed the data, and wrote the manuscript. MC, CA, and ML performed the research. J-CG and SB performed the statistical analysis, analyzed the data, and wrote the manuscript. TB performed the extraction from the national “Programme de médicalisation des systèmes d’information” (PMSI) database. All authors contributed to the article and approved the submitted version.

## References

[1] Tang N Li D Wang X Sun Z. Abnormal coagulation parameters are associated with poor prognosis in patients with novel coronavirus pneumonia. *J Thromb Haemost.* (2020) 18:844–7. 10.1111/jth.14768 32073213 PMC7166509

[2] Al-Samkari H Karp Leaf R Dzik W Carlson J Fogerty A Waheed A COVID-19 and coagulation: bleeding and thrombotic manifestations of SARS-CoV-2 infection. *Blood.* (2020) 136:489–500. 10.1182/blood.2020006520 32492712 PMC7378457

[3] Connors J Levy J. Thromboinflammation and the hypercoagulability of COVID-19. *J Thromb Haemost.* (2020) 18:1559–61. 10.1111/jth.14849 32302453 PMC9770920

[4] Bian XW. COVID-19 pathology team. Autopsy of COVID-19 patients in China. *Natl Sci Rev.* (2020) 7:1414–8. 10.1093/nsr/nwaa123 34192086 PMC7313767

[5] Brinkmann V Reichard U Goosmann C Fauler B Uhlemann Y Weiss D Neutrophil extracellular traps kill bacteria. *Science.* (2004) 303:1532-5. 15001782 10.1126/science.1092385

[6] Middleton E He X Denorme F Campbell R Ng D Salvatore S Neutrophil extracellular traps contribute to immunothrombosis in COVID-19 acute respiratory distress syndrome. *Blood.* (2020) 136:1169–79. 10.1182/blood.2020007008 32597954 PMC7472714

[7] Prével R Dupont A Labrouche-Colomer S Garcia G Dewitte A Rauch A Plasma markers of neutrophil extracellular trap are linked to survival but not to pulmonary embolism in COVID-19-Related ARDS patients. *Front Immunol.* (2022) 13:851497. 10.3389/fimmu.2022.851497 35371025 PMC8968169

[8] Matsumoto H. The technology of reagents in the automated hematology analyzers Sysmex XE-2100™ - red fluorescence technology. *Sysmex J Int.* (1999) 9:179185.

[9] Stiel L Delabranche X Galoisy A Severac F Toti F Mauvieux L Neutrophil fluorescence: a new indicator of cell activation during septic shock-induced disseminated intravascular coagulation. *Crit Care Med.* (2016) 44:e1132–6. 27441905 10.1097/CCM.0000000000001851

[10] Stiel L Mayeur-Rousse C Helms J Meziani F Mauvieux L. First visualization of circulating neutrophil extracellular traps using cell fluorescence during human septic shock-induced disseminated intravascular coagulation. *Thromb Res.* (2019) 183:153–8. 31678710 10.1016/j.thromres.2019.09.036

[11] Delabranche X Stiel L Severac F Galoisy A Mauvieux L Zobairi F Evidence of NETosis in septic shock induced disseminated intravascular coagulation. *Shock.* (2017) 47:313–7. 27488091 10.1097/SHK.0000000000000719

[12] Stiel L Rabouel Y Debliquis A Pointurier V Mootien J Kuteifan K. NEUT-SFL in Patients with COVID-ARDS: a novel biomarker for thrombotic events? *Dis Markers.* (2021) 2021:4361844. 10.1155/2021/4361844 34840629 PMC8612800

[13] Gris J Cochery-Nouvellon É Bourguignon C Mercier É Bouvier S Quéré I Reference values of coagulation assays performed for thrombophilia screening after a first venous thrombosis and their intra-patient associations. *Thromb Res.* (2022) 210:94–103. 10.1016/j.thromres.2022.01.005 35042062

[14] Bouvier S Bastide S Chouirfa S Nouvellon É Mercier É Bigot L Reliability of hemostasis biomarkers is affected by time-dependent intra-patient variability. *J Thromb Haemost.* (2018). 10.1111/jth.14198 [Epub ahead of print]. 29883046

[15] Zhu Y Chen X Liu X. NETosis and neutrophil extracellular traps in COVID-19: immunothrombosis and beyond. *Front Immunol.* (2022) 13:838011. 10.3389/fimmu.2022.838011 35309344 PMC8924116

[16] Arachchillage D Laffan M. Abnormal coagulation parameters are associated with poor prognosis in patients with novel coronavirus pneumonia. *J Thromb Haemost.* (2020) 18:1233–4. 10.1111/jth.14820 32291954 PMC7262191

[17] Dennison D Al Khabori M Al Mamari S Aurelio A Al Hinai H Al Maamari K Circulating activated neutrophils in COVID-19: an independent predictor for mechanical ventilation and death. *Int J Infect Dis.* (2021) 106:155–9. 10.1016/j.ijid.2021.03.066 33781906 PMC7997692

[18] Mohammad N Nazli R Zafar H Fatima S. Effects of lipid based multiple micronutrients supplement on the birth outcome of underweight pre-eclamptic women: a randomized clinical trial. *Pak J Med Sci.* (2022) 38:219–26. 10.12669/pjms.38.1.4396 35035429 PMC8713215

[19] Masso-Silva J Moshensky A Lam M Odish M Patel A Xu L Increased peripheral blood neutrophil activation phenotypes and neutrophil extracellular trap formation in critically Ill Coronavirus disease 2019 (COVID-19) patients: a case series and review of the literature. clin infect dis. 2022 74(3):479-489. erratum in. *Clin Infect Dis.* (2022) 74:1889–90. 10.1093/cid/ciab437 33988226 PMC8241438

[20] Henry B de Oliveira M Benoit S Plebani M Lippi G. Hematologic, biochemical and immune biomarker abnormalities associated with severe illness and mortality in coronavirus disease 2019 (COVID-19): a meta-analysis. *Clin Chem Lab Med.* (2020) 58:1021–8. 10.1515/cclm-2020-0369 32286245

[21] Gao Y Ding M Dong X Zhang J Kursat Azkur A Azkur D Risk factors for severe and critically ill COVID-19 patients: a review. *Allergy.* (2021) 76:428–55. 10.1111/all.14657 33185910

[22] Dessie Z Zewotir T. Mortality-related risk factors of COVID-19: a systematic review and meta-analysis of 42 studies and 423,117 patients. *BMC Infect Dis.* (2021) 21:855. 10.1186/s12879-021-06536-3 34418980 PMC8380115

